# Implant-Retained Mandibular Overdentures: Patient-Related Outcome Measurements after Seven Years of Function

**DOI:** 10.3390/dj10050088

**Published:** 2022-05-16

**Authors:** Jan D'haese, Carine Matthys, Hamed Sahak, Jos Besseler, Hugo De Bruyn

**Affiliations:** 1 Department of Dentistry, Radboud University Medical Center, Radboud Institute for Health Sciences, 6525 EX Nijmegen, The Netherlands; hamedsahak@gmail.com (H.S.); hugo.debruyn@radboudumc.nl (H.D.B.); 2 Department Periodontology & Oral Implantology, Dental School, Faculty Medicine and Health Sciences, Ghent University, 9000 Ghent, Belgium; carine.matthys@ugent.be; 3 Besseler Dental Clinic, 7514 DZ Enschede, The Netherlands; mail@josbesseler.nl

**Keywords:** maintenance cost, patient related outcome measurements, quality of life, mandibular overdentures, edentulism

## Abstract

Denture wearers often complain about jeopardized function and reduced quality of life due to lack of prosthesis’ retention. Implant-retained mandibular overdentures, on two non-connected implants (2IOD) are well-proven solutions to overcome these issues. We prospectively assessed 69 patients and scrutinized clinical records until at least seven years of function. Thirty-six were retained on Locator^®^ Abutments (LA) and thirty-tree on Ball Abutments (BA). Both systems were compared regarding the type, amount, and total cost of required maintenance. One implant was lost, yielding 98.7% survival after seven years. In total, 438 technical issues occurred: 121 (27.35%) in BA and 317 (72.4%) in LA. Out of these, 343 events (78%) were solved chairside: 191 (43.6%) were replacements of retention caps, 113 (25.8%) were minor acrylic repairs, 26 (5.9%) pressure ulcers had to be relieved, and 13 (3%) were related to abutments. LA required 179 insert replacements compared to 12 in the BA group. The overall initial treatment cost was EUR 3850 (base year of the analysis: 2003). The average total maintenance cost in relation to the initial cost for the LA and BA groups was 19.11 (range 0–82.24%) and 18.91% (range 0–113.26%) respectively (*p* = 0.540). Conclusions: The seven-year maintenance costs for a 2IOD is acceptable when the patient is regularly checked and professionally maintained. Most events are easily solvable chairside, but a few patients required more expensive interventions, regardless of the type of attachment used.

## 1. Introduction

An important indication for considering dental implant placement is dysfunction of an existing partial or complete dental prosthesis [1]. In edentulous patients, it is a known phenomenon that retention and stability of a lower removable prosthesis decreases due to ongoing resorption of the jawbone. Therefore, an implant-retained overdenture on two non-connected implants (2IOD) by means of a Locator^®^ Abutment (LA) or Ball Abutments (BA) or connected implants with a bar suprastructure can be helpful to provide extra stability and retention [1]. Several studies were published on the 2IOD’s in the mandible. Most of them reported on Patient-Related Outcome Measurements (PROM) such as the Oral Health Impact Scale (OHIP), the McGill Patient Satisfaction Questionnaire, Denture Satisfaction Questionnaire, or the Oral Impacts on Daily Performances [2]. The systematic review of Kutkut and coworkers [2] investigated how a conventional removable prosthesis performs compared to an 2IOD based on PROM’s, and concluded that overdentures gave better results than conventional prostheses. Visser and coworkers [3] showed that maintenance for mandibular overdentures on implants was most of the time limited to minor adjustments of the prosthesis in order to release pressure ulcers, providing oral hygiene instructions and removing calculus. Fromentin and coworkers [4] investigated the amount of wear of the matrices in mandibular overdentures supported by ball abutments. A total of 35 patients (70 matrices) were evaluated after one year (*n* = 26), three years (*n* = 28), and eight years (*n* = 16). The authors concluded that after one year there has been virtually no change in dimension and deformation. However, between one and three years, a significant amount of wear was observed on the matrices. In seven patients, one of the matrices had to be replaced and even in five patients this involved replacement of both matrices. Stoker and coworkers [5] compared three different kinds of suprastructures (bar construction on four implants, bar construction on two implants, and two implants with ball abutments) used for mandibular overdentures regarding maintenance costs. They concluded that most aftercare was needed in the ball abutments group, in particular related to retention loss. The maintenance cost on average was USD 997.43 ± 620.2 up to eight years of function. The aim of the present study was firstly to report on the seven-year implant survival, secondly to evaluate the amount of maintenance in terms of technical repairs, and thirdly to calculate the maintenance cost involved for two-implant retained overdentures in the mandible after seven years of function, respectively retained by Locator ^®^ or Ball Abutments.

## 2. Experimental Section

### 2.1. Study Design and Surgical Procedure

This clinical cohort study was performed in a dental clinic in Enschede, the Netherlands. Only completely edentulous individuals in need of an implant-retained prosthesis were consecutively treated by one surgeon/prosthodontist (JB) between 2003 and 2013. The participants were healthy, without cognitive impairment and without a medical history of radiotherapy in the oral cavity. All patients received two bone level implants (Osseospeed, Dentsply Sirona, Molndal, Sweden) placed in a one-stage procedure. Postoperatively, the prosthesis was relined and adapted, allowing a nonfunctional implant integration for three months. After confirmation of successful integration, the implants were provided with either ball or locator abutments. For the details concerning patient selection and surgical and prosthetic treatment, we refer to the previous published article by Matthys and coworkers [6]. The treatment groups were not randomized. The two cohorts were treated according to the system used in the clinic at the moment of implant placement. The surgical procedure and the fabrication of the prosthesis were done by the same dentist (J.B.).

### 2.2. Study Population

**Figure 1:**
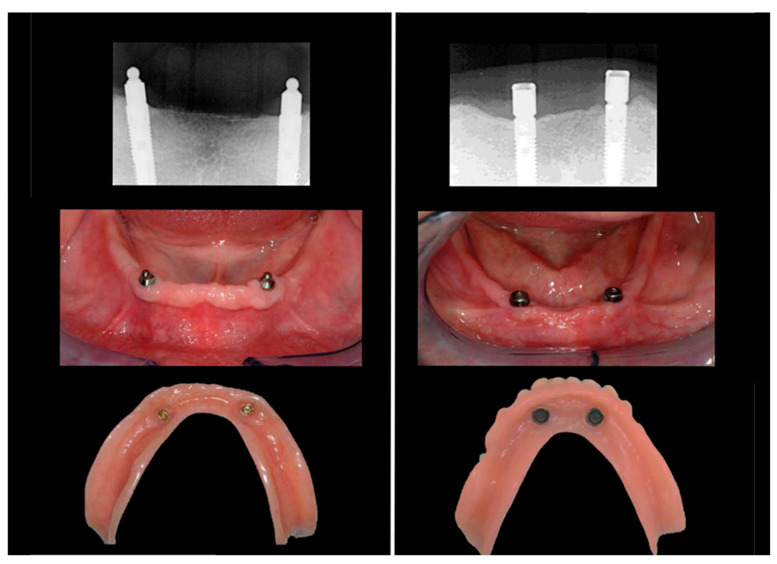
Radiographical and clinical example an overdenture retained on 2 non-connected implants on either 2 ball abutments (left) or 2 locator abutments (right).

Only fully edentulous patients wearing a removable upper and lower prosthesis were eligible for the assessment. The lower prosthesis had to be an overdenture retained on two non-connected implants on either two locator or two ball abutments (Figure 1). Patients who received implants in the maxilla in the course of the follow-up were excluded from the analysis and considered as protocol deviations. Only patients with at least a seven-year follow-up were selected. Smokers were also included.

### 2.3. Research Variables

Throughout annual clinical research examinations, an independent team of calibrated prosthodontists and periodontists from the Department of Periodontology and Prosthodontics at the Ghent University, Belgium, collected the research data.

The following variables of the selected patients were recorded regarding surgical procedure and clinical outcome: date of implant surgery, date of abutment connection, type of abutment used, and implant survival.

In addition, the following information was retrospectively collected from the clinical records: amount and costs of maintenance, amount and cost of repairs, and finally, amount and costs of component replacement related to the implants and the suprastructures.

The study analyzed the cost from a patient’s point of view, regardless of who paid for the costs (patient or health care insurance). The initial treatment cost was registered and used as a reference. This included presurgical planning, implant surgery including material cost and dentist fee, and prosthetical cost including all materials, dentist, and technician fees. The maintenance cost per year was recorded (including all materials and fees). This included repair of upper and lower dentures, all interventions and replacements of the retention systems, new implants if needed, new dentures, follow-up consultations, and small interventions such as professional cleaning of the dentures in the lab and retightening of an abutment. The cost for annual peri-implant health maintenance was not taken into account because it was not an outcome measurement and because it was regardless of the attachment system used (BA or LA). Indirect costs for the patient (travel time, opportunity cost) were not taken into consideration as the population being analyzed lives in the neighborhood of the dental centers and most patients are retired. No clinicians’ costs were applied either because the focus of the study was on the patient’s perspective, rather than analyzing the profit for the clinician.

### 2.4. Statistical Analysis

Statistical analysis was performed using SPSS Statistics 25 (IBM Armonk, NY, USA). Descriptive statistics were used to report the total patient group. The Mann-Whitney U test was used to compare costs of maintenance and repair as these are ordinal variables. The level of significance was set at *p* < 0.05.

The study was approved by the ethical committee of the Ghent University Hospital (EC UZ 2005/414). All participants gave written consent.

## 3. Results

### 3.1. Clinical Outcome

**Figure 2:**
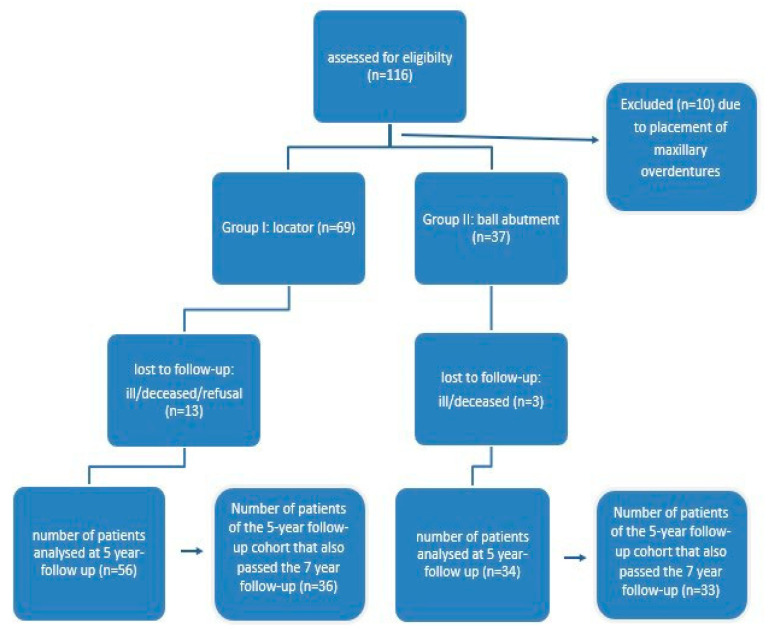
Decision tree used to select the cases to be assessed.

A total of 69 fully edentate patients (male: 41, female: 28) originating from 90 investigated earlier on [6,7] also passed the seven-year follow up and have been included in this study (Figure 2). Mean age at time of surgery was 65 years (range 46–83). Out of the 69 included patients, ten were current smokers (14.49%). Table 1 gives an overview of all the characteristics of the patients included.

One of the implants was lost during follow-up, leading to an overall implant survival of 98.7% up to seven years after implant insertion. No specific biological complications were reported.

### 3.2. Maintenance

Table 2 shows the total amount of maintenance performed up to seven years for the whole group and for each of the treatment groups separate. A total of 438 technical or maintenance issues occurred in the 69 patients in seven years; respectively, 121 (27.35%) in BA and 317 (72.4%) in LA. The vast majority (343/438 = 78%) were minor technical problems that could easily be solved chairside. This included 191 (43.6%) replacements of retention caps, 113 (25.8%) minor acrylic repairs, 26 (5.9%) relieve of pressure ulcera, and 13 (3%) related to abutments. Only a minority (95/438 = 22%) required repair by the dental technician. This included 77 (17.6%) rebasing procedures, 13 (3%) involved production of a new denture, and 5 (1%) were extensive cleaning procedures to remove staining and calculus.

### 3.3. Costs

Replacements of abutments represented the highest amount of costs. Sometimes, the manufacturing of new prosthesis was required.

**Figure 3:**
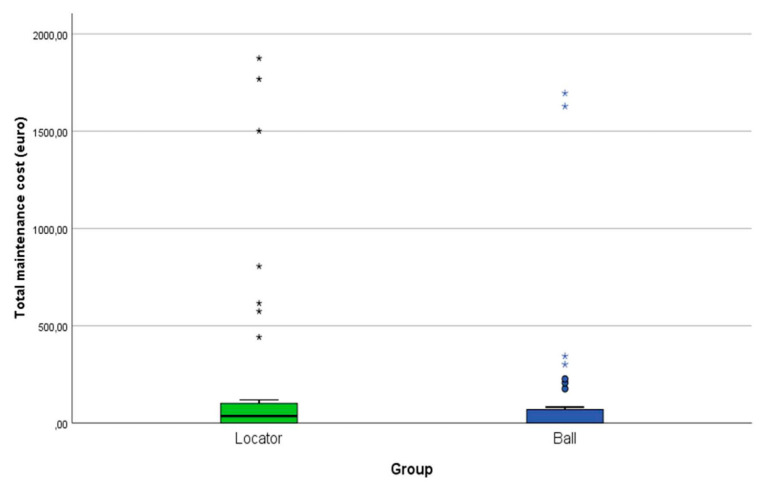
Cumulative amount of maintenance costs in relation to the initial treatment costs. No significant difference observed between both treatment groups (*p* = 0.540). * and o represent the outliners.

**Figure 4:**
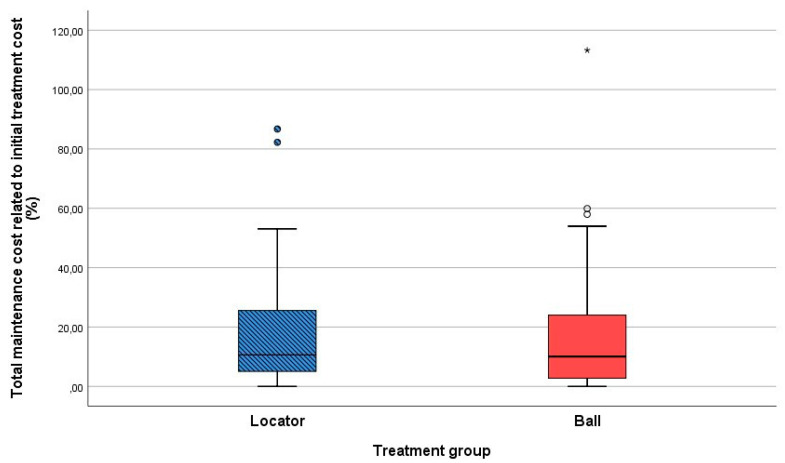
Maintenance costs up to seven years in function in relation to the initial treatment cost for both treatment groups. * and o represent the outliners.

We used the year 2003 as the base year for the economic analysis. The average initial cost for the treatment was EUR 3850 [6,7]. When comparing the cumulative amount of maintenance costs in relation to the initial treatment costs, there was no significant difference (*p* = 0.540) between both treatment groups (Figure 3). The mean maintenance costs after seven years amounted to 19.11% (EUR 735.75) [0–82.2% range] and 18.91% (EUR 728.04) [0–113.26% range] of the initial treatment cost for the locator and ball attachment group, respectively (Figure 4).

## 4. Discussion

In the present study, we examined a cohort of patients who have been carefully followed for at least seven years. All patients were treated in a private practice and were frequently recalled for maintenance and aftercare. All events were carefully documented in the patients’ files, providing a realistic overview of the total amount of aftercare needed. We specifically choose to include all treated subjects because this reflects the normal condition in daily practice and to avoid a distorted picture in the outcome. Previous research on cost and effect of 2IOD (ball and bar) constructions reports a gain in oral outcome effect in comparison with conventional dentures, provided the patient is willing to pay a certain price. Comparison between studies is often difficult for various reasons, the most important being the implementation of indirect participants cost, the extrapolation of cost or effects in future years, and variations in dentist fees among countries (Alfadda and Attard, 2017; Heydecke et al., 2005; Zitzmann et al., 2006). The study of Alfadda [8] on 2IOD comparing two loading protocols on bar/clip attachment and inclusion of the indirect participants cost reports an overall cost of CAD 255.60 per point OHIP-20 improvement at the denture insertion and CAD 170.58, CAD 210.38, and CAD 478.70 at the 1-, 5- and 14-year follow-ups, respectively. The research of Zitzmann et al. (2013) [9] compared conventional dentures and two types of implant overdentures (two and four implants). In a three-year analysis, the cost per quality-adjusted prosthesis year gained with implant overdentures is CHF 9100 for the two implants’ overdenture on Ball abutments and CHF 19,800 for the four implant bar overdenture. The ten-year extrapolation reports values of CHF 3800 for the two implants ball and CHF 7100 for the four implant bar overdenture. In the work of Heydecke et al. (2005) [10], conventional dentures and two IOD on ball attachments were analyzed. The one-year results comparing cost (direct and indirect) and health effect in OHIP-20 points were the basis for an extrapolation up to 17 years, reporting a supplementary cost for 2IOD treatment of CAD 14.41 per OHIP-20 point per year.

Our retrospective analysis evaluated the cost, regardless of the fact who eventually paid for it. In the Netherlands, the 2IOD in the mandible is included in the basic health care coverage and patients only pay around 15% of the surgical and technical fee out of their own budget. In order to evaluate maintenance cost, we intended to describe these costs in relation to the initial treatment cost. The initial treatment cost was taken as a baseline and the total technical maintenance cost per year up to seven years was expressed as a percentage of the initial treatment cost. One should, however, consider that inflation is also a relevant factor when comparing costs, as it introduces a bias when comparing maintenance costs over time between participants. The participants analyzed in this article have received their 2IOD between 2003 and 2013. Maintenance costs are logically spoken and only applicable after installation of the 2IODs. As there was never negative inflation in the Netherlands from 2003 (first patient enrolled) on [11], expressing the percentual maintenance cost in relation to the initial treatment cost results in an overestimation. So the actual percentual maintenance cost should even be lower in reality to the ones stated in our manuscript.

Only a limited amount of studies were found that specifically scrutinized technical aftercare with a minimum follow-up of five years. Farsai and coworkers [12] concluded that, on average, after 12 years of function, the overdentures had to be replaced. Furthermore, the cost of aftercare was on average USD 1400,—up to ten years. However, there was a wide individual variation [12].

The data obtained from our patient records clearly demonstrated the excellent clinical outcome of the intra-osseous implants as only one implant had been lost up to seven years in function. This is in accordance with Matthys and coworkers [7], who also concluded that overdentures on implants have a high survival rate. Our results showed that when we look at the entire population, costs slightly increase slightly over the years, although this is not statistically significant.

Krennmair et al. [13] compared mandibular overdentures retained on ball or telescopic crown attachments in a group of 25 patients and reported on implant success, peri-implant conditions, and subjective patient satisfaction. Scores did not differ between the two retention modalities used. However, during the five-year observation period, significantly more (*p* < 0.01) postinsertion complications/interventions for maintenance purposes were registered in the ball group (87 interventions, 61.1%) than in the telescopic crown group (53 interventions, 37.9%). Differences in prosthodontic maintenance were most significant in the second and third years (*p* < 0.05) of the follow- up period, but were similar at the end of the study for both anchorage systems.

Zhang et al. [14] reported that a mandibular IOD was a beneficial treatment option for seniors with a history of deficient complete dentures, improving denture-quality, patient satisfaction, and reducing patient complaints up to five years. Maintenance events clustered on the first year, showing no significant impact on long-term patient satisfaction and other PROMs.

Bakker et al. [15] published a twenty-year follow-up paper on 15 patients. The twenty-year implant survival rate was 92.5%. Radiographic analysis revealed minor marginal bone loss during the first ten years and no further loss thereafter. Participants were very satisfied with their prosthesis and reported a good quality of life. At the twenty-year evaluation, 64.3% of the patients were classified as frail.

From the five-year follow-up time point on, the number of repairs became slowly higher (although not significant) compared to the first years because of technical interventions mainly due to the manufacturing of some new prosthesis or replacement of abutments for wear and tear.

**Figure 5:**
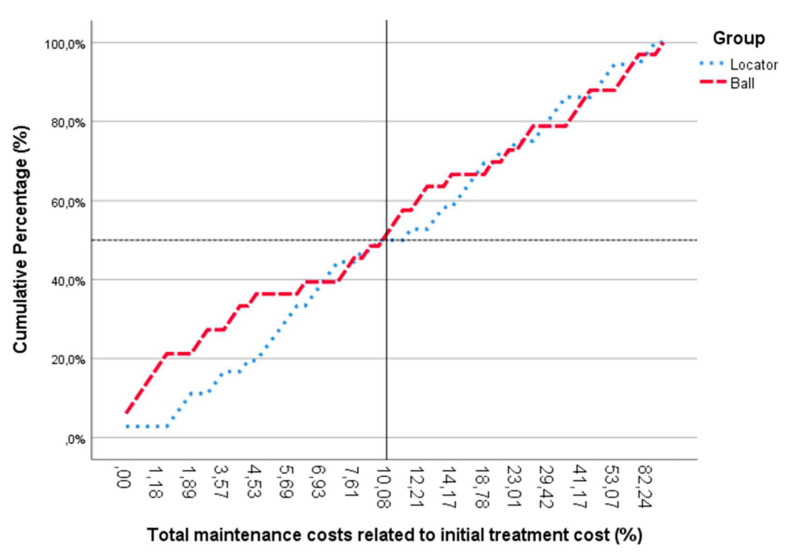
Cumulative percentage of maintenance costs compared to the initial treatment cost. For 50% of the total population, the amount of total maintenance cost is around 10% compared to the initial cost.

Looking at the cumulative percentage of maintenance costs, compared to the initial treatment cost (Figure 5), it can be seen that the maintenance costs up to seven years for 50% of the total population is around 10% compared to the initial cost.

These costs are relatively low in dental care, as most public health insurers reimburse a substantial amount of the costs made. Remarkably, there is a large difference in the amount of consultations between the locator and the ball abutment groups (respectively, 179 versus 12) regarding issues with the retention components (Table 2). This is related to the composition of the components of both retention systems that differ from each other. The locator abutment consists of a small plastic part (matrix) that wears out faster and needs more often to be replaced. The ball abutment does not contain plastic parts. It only consists of metal parts that wear out less quickly. The cost of replacing these small plastic matrices is quite low, so this does not quite increase the maintenance costs. However, one has to keep in mind that patients with locator abutments more often need to consult the dental office for maintenance related to retention issues. One may speculate on the consequence of this difference when patients are not properly maintained on a yearly basis. When the inserts are worn out, misfit leading to direct contact with the locator may lead to abutment damage. This may be a consequence with detrimental effects, especially in aging and more fragile patients that are not always able to attend regular recall maintenance.

## 5. Conclusions

In a well-maintained population, the mandibular 2IOD restoration yield nearly 98% of implant survival. Technical complications regularly occur, although are mostly easy to repair. On a population level, the amount of maintenance cost up to seven years is proportionally less than 20% of the initial treatment cost, irrespective of type of retention. However, there is a big range due to some outliers.

## Figures and Tables

**Table 1:** Overview of the different treatment groups regarding distribution, age, and smoking habits.

	Group 2: Ball Abutment	Group 1: Locator Abutment
Total number of patients (male/female)	36 (20/16)	33 (21/12)
Age at implant insertion (range)	65 (49-80)	64 (46-83)
Number of smokers	7	3

**Table 2:** Detailed specification of all maintenance issues and repairs between the different treatment groups (B = Ball, L = Locator) up to 7 year after implant insertion.

	Minor Acrylic Repairs		Rebasing Overdenture		Mucosal Pressure Ulcera		Retention Issues		Abutment Loosening		Abutment Replacement		Overdenture Cleaning		Overdenture Replacement		Total
	B	L	B	L	B	L	B	L	B	L	B	L	B	L	B	L	
Year 1	6	2	8	4	4	0	10	0	0	0	0	0	0	0	0	0	34
Year 2	7	8	8	7	4	2	3	3	0	0	0	0	1	0	0	0	43
Year 3	8	14	5	4	3	0	27	3	0	0	1	1	0	0	0	2	68
Year 4	8	9	2	6	1	0	21	1	1	1	1	0	0	0	0	0	51
Year 5	11	9	6	5	4	1	32	1	0	0	0	0	1	1	3	0	74
Year 6	10	8	5	9	2	1	40	1	0	0	3	0	2	0	0	3	84
Year 7	10	3	3	5	3	1	46	3	0	0	4	1	0	0	3	2	84
Total	60	53	37	40	21	5	179	12	1	1	9	2	4	1	6	7	438

## Data Availability

The data presented in this study are available on request from the corresponding author.
